# Rhesus Monkeys (*Macaca mulatta*) Sense Isochrony in Rhythm, but Not the Beat: Additional Support for the Gradual Audiomotor Evolution Hypothesis

**DOI:** 10.3389/fnins.2018.00475

**Published:** 2018-07-16

**Authors:** Henkjan Honing, Fleur L. Bouwer, Luis Prado, Hugo Merchant

**Affiliations:** ^1^Amsterdam Brain and Cognition, Institute for Advanced Study, Institute for Logic, Language and Computation, University of Amsterdam, Amsterdam, Netherlands; ^2^Department of Cognitive Neuroscience, Instituto de Neurobiología, Universidad Nacional Autonoma de México, Santiago de Querétaro, Mexico

**Keywords:** music, rhythm, beat perception, ERP, MMN

## Abstract

Charles Darwin suggested the perception of rhythm to be common to all animals. While only recently experimental research is finding some support for this claim, there are also aspects of rhythm cognition that appear to be species-specific, such as the capability to perceive a regular pulse (or beat) in a varying rhythm. In the current study, using EEG, we adapted an auditory oddball paradigm that allows for disentangling the contributions of beat perception and isochrony to the temporal predictability of the stimulus. We presented two rhesus monkeys (*Macaca mulatta*) with a rhythmic sequence in two versions: an isochronous version, that was acoustically accented such that it could induce a duple meter (like a march), and a jittered version using the same acoustically accented sequence but that was presented in a randomly timed fashion, as such disabling beat induction. The results reveal that monkeys are sensitive to the isochrony of the stimulus, but not its metrical structure. The MMN was influenced by the isochrony of the stimulus, resulting in a larger MMN in the isochronous as opposed to the jittered condition. However, the MMN for both monkeys showed no interaction between metrical position and isochrony. So, while the monkey brain appears to be sensitive to the isochrony of the stimulus, we find no evidence in support of beat perception. We discuss these results in the context of the gradual audiomotor evolution (GAE) hypothesis (Merchant and Honing, 2014) that suggests beat-based timing to be omnipresent in humans but only weakly so or absent in non-human primates.

## Introduction

The interest in rhythm cognition in non-human animals is motivated by the search for signs of *musicality* as a means to get an insight in the evolutionary and causal processes underlying human musicality (Trehub, 2003; Honing and Ploeger, 2012; Hoeschele et al., 2015; Honing et al., 2015; Trehub et al., 2015; Honing, 2018b)^1^. Most animals show at least some sort of rhythmic behavior, like walking, flying, crawling, or swimming. It is hence not unnatural to think that the perception (and enjoyment) of rhythm might be shared by most animals, as was argued by Darwin (1871) and Patel (2014). While only recently experimental research is finding some support for this claim (Wilson and Cook, 2016), there are also aspects of rhythm cognition that appear to be species-specific (Fitch, 2013), such as the capability to perceive a regular pulse in a varying rhythm (i.e., one level of a metrical structure) and consequently being able to synchronize to it (i.e., rhythmic entrainment), referred to as *beat-based timing* (Merchant and Honing, 2014).

Beat-based timing in humans is a complex neurocognitive phenomenon that depends on a dynamic interaction between auditory and motor systems in the brain (Grahn and Brett, 2009; Morillon et al., 2014; Patel and Iversen, 2014; Hoeschele et al., 2015; Merchant and Yarrow, 2016; Ross et al., 2017). It is hypothesized to be facilitated by bidirectional, and potentially causal links between the auditory and motor areas in the brain, including the motor cortico-basal-ganglia-thalamo-cortical (mCBGT) circuit, that appear to be more developed in humans as opposed to non-human primates and related species (Patel et al., 2009; Mendoza and Merchant, 2014; Patel and Iversen, 2014; Petkov and Jarvis, 2014; Merchant et al., 2015a; Wilson and Cook, 2016).

These observations lead to the *gradual audiomotor evolution (GAE)* hypothesis (Merchant and Honing, 2014) that suggests beat-based timing to be gradually developed in primates, peaking in humans but present only with limited properties in other non-human primates, while humans share interval-based timing with all non-human primates and related species. Thus, the GAE hypothesis accommodates the fact that the performance of rhesus monkeys is comparable to humans in single interval tasks—such as categorization, interval reproduction, and interception—, but differs in multiple interval tasks, such as synchronization, continuation, and rhythmic entrainment (Honing and Merchant, 2014; Merchant and Honing, 2014).

In the current paper, we will focus on beat and isochrony perception as two key components of musicality (Merchant et al., 2015b; Honing, 2018a), and provide further evidence for the GAE hypothesis by studying rhythm perception in two rhesus monkeys (*Macaca mulatta*). For this study we used an existing ERP paradigm that allows for testing and disentangling the contributions of beat perception and isochrony to the temporal predictability of the stimulus (Bouwer et al., 2016).

## Earlier work

Most existing animal studies on beat-based-timing and rhythmic entrainment (Wilson and Cook, 2016) have used behavioral methods to probe the presence of beat perception, such as tapping tasks (Zarco et al., 2009; Hasegawa et al., 2011; Hattori et al., 2015) or measuring head bobs (Patel et al., 2009; Schachner et al., 2009; Cook et al., 2013). However, if the production of synchronized movement to sound or music is not observed in certain species, this is no evidence for the absence of beat perception. It could well be that while certain species are not able to synchronize their movements to a regular beat, they may be capable of *beat perception* (i.e., the ability to perceive a regular pulse in a temporally and/or acoustically varying rhythm; Honing, 2012). With behavioral methods that rely on overt motoric responses it is difficult to separate between the contribution of perception and action. More direct, electrophysiological measures such as auditory event-related brain potentials (ERPs) allow to test for neural correlates of rhythm cognition, including beat perception (Honing et al., 2014).

While the vast majority of previous studies on animals have used implanted electrodes to record electroencephalograms (EEG) (Javitt et al., 1994; Laughlin et al., 1999; Pincze et al., 2001), non-invasive electrophysiological techniques such as scalp recorded evoked potentials (EP) and event-related potentials (ERP) are considered an attractive alternative. Next to being a mandatory requirement for studying some non-human primates such as chimpanzees (Fukushima et al., 2010; Hirata et al., 2013), these methods allow for a direct comparison between human and non-human primates. As such they have contributed to establishing animal models of the human brain and human brain disorders (Godlove et al., 2011; Gil-da-Costa et al., 2013), a better understanding of the neural mechanisms underlying the generation of human evoked EP/ERP components (Fishman and Steinschneider, 2012), as well as delineating cross-species commonalities and differences in brain functions, including rhythm cognition (Ueno et al., 2008, 2010; Fukushima et al., 2010; Reinhart et al., 2012; Hirata et al., 2013; Itoh et al., 2015). We will describe the most relevant ERP components for rhythm perception below.

## Using ERPs in measuring beat perception

The mismatch negativity (MMN) is an auditory event-related component that was shown to be sensitive to rhythmic violations in both humans and monkeys (see Honing et al., 2014 for a review). The MMN can be used as an index of a violation of temporal expectation using an oddball paradigm, by identifying a negative peak shortly after the deviant (the “oddball”) that is maximal at fronto-central midline electrode sites and has sources in the auditory cortices and in the inferior frontal gyrus (i.e., not primarily reflecting motor cortex activity; Gil-da-Costa et al., 2013). The larger the violation of rhythmic expectations, the larger is the amplitude of the MMN (Näätänen et al., 2007; Winkler, 2007). The MMN has been shown to be indicative of beat perception in humans, with deviants on the beat within a repeating metrical auditory pattern eliciting a larger MMN than deviants off the beat (Ladinig et al., 2009, 2011; Winkler et al., 2009; Bouwer et al., 2014, 2016; Honing et al., 2014; Bouwer and Honing, 2015; Mathias et al., 2016).

The P3a, thought to reflect the redirection of attention to a deviant stimulus (Polich, 2007) and possibly an index of the conscious perception of a deviant (cf. Mathias et al., 2016; Peretz, 2016), often emerges just after the MMN, and has a latency of 200–250 ms in humans and between 100 and 250 ms in rhesus monkeys (Picton et al., 1974; Polich, 2007). Gil-da-Costa et al. (2013) provided functional evidence that the neural generators of both MMN and P3a may be homologous in humans and monkeys, despite the observed differences in latency (see Table 1 for an overview).

**Table 1:** Homologies between rhesus monkey, chimpanzee, and human cortical auditory evoked potentials (ERPs).

	**Human scalp Picton et al**.	**Ape scalp Ueno et al**.	**Monkey scalp Itoh et al**.	**Monkey scalp Gil-da-Costa et al**.	**Monkey scalp Honing et al**.	**Monkey cranial Teichert**	**Monkey epidural Javitt et al**.
P1	50–60		25–30 [mP1]	–	–	45–65 [P55]	5–40
N1	75–100		45–65 [mN1]	–	–	70–105 [N85]	40–120
MMN	100–200	125–180	–	48–120	60–110	–	–
P3a	200–250		–	100–250	–	–	–

*Time range in ms; alternative naming in square brackets (Adapted from Picton et al., 1974; Javitt et al., 2000; Ueno et al., 2008; Honing et al., 2012; Gil-da-Costa et al., 2013; Itoh et al., 2015; Teichert, 2016)*.

In addition to the ERP responses that reflect the detection of a deviant stimulus, the P1 and N1 responses, two early auditory event-related components, have been shown to be sensitive to the timing of the stimulus presentation (Costa-Faidella et al., 2011; Schwartze et al., 2013; Pereira et al., 2014; Teichert, 2016). In general, both P1 and N1 have an inverse relationship of ERP amplitude with temporal predictability (Javitt et al., 2000; Costa-Faidella et al., 2011; Schwartze et al., 2013). As such they are not indicative of beat perception *per se*, but of the temporal predictability of the stimulus. For instance, Schwartze et al. (2013) showed the P1 and N1 to be smaller in an isochronous as opposed to a jittered rhythmic sequence. Generally, increasing the predictability of the auditory stimulation (both in stimulus timing and stimulus probability) leads to a pronounced N1 attenuation (Costa-Faidella et al., 2011). Both components are maximal over fronto-central electrodes and there is some consensus on their homolog in rhesus monkeys, most notably on the N1 (Teichert, 2016) (see Table 1 for an overview).

## Rhythm cognition in monkeys

Recently, Honing et al. (2012) were able to show, for the first time, that an MMN-like response can be measured in rhesus monkeys (*M. mulatta*). [See (Ueno et al., 2008) using a similar method in a chimpanzee (*Pan troglodytes)*, and (Gil-da-Costa et al., 2013) for a recent study comparing humans and macaques (*Macaca fascicularis*)].

In addition, Honing et al. (2012) showed a sensitivity of the MMN in response to pitch deviants and infrequent omissions, showing that it was possible, in principle, to use an identical paradigm for human and non-human primates to probe beat perception. However, and contrary to what was found in human adults and infants (Winkler et al., 2009; Bouwer et al., 2014), no difference was found in the MMN in response to omissions in beat and offbeat positions. This lead to the conclusion that rhesus monkeys are unable to sense the beat (Honing et al., 2012). In addition, a strong response was found for onsets of rhythmic groups suggesting a sensitivity to rhythmic structure (A similar result was reported in Selezneva et al., 2013 showing large responses to changes in a repeating temporal pattern, while measuring gaze and facial expressions in monkeys).

However, Bouwer et al. (2014) pointed out that the earlier paradigm (Winkler et al., 2009; Honing et al., 2012) needs additional controls to be certain that any effects (or the lack thereof) are due to beat perception, and not, for instance, a result of pattern matching, acoustic variability or sequential learning. While rhesus monkeys have apparently little or no ability to perceive a beat, they are able to detect the regularity of an isochronous visual or auditory metronome (Zarco et al., 2009; Merchant et al., 2015b; Gámez et al., 2018). This suggests a capacity for making temporal predictions which most likely depends on absolute interval perception (Merchant and Honing, 2014). As such monkeys might not have beat perception, but could still be able to sense the regularity in an isochronous stimulus.

To examine the perception of isochrony, several studies have compared the responses to temporally regular, isochronous sequences with the responses to temporally irregular, jittered sequences (Schwartze et al., 2011; Teki et al., 2011; Fujioka et al., 2012). The prediction of events in jittered sequences has been suggested to rely on absolute interval perception, while the prediction of events in isochronous sequences has been suggested to be based on beat perception (Schwartze et al., 2011; Fujioka et al., 2012). However, it is possible to predict events in isochronous sequences on the basis of absolute interval perception alone, which may explain why macaques, with little or no ability to perceive a beat (Honing et al., 2012; Merchant and Honing, 2014), respond more accurately to temporally regular than jittered sequences (Zarco et al., 2009), based on their isochrony, rather than on beat-based perception (Merchant et al., 2015a; Merchant and Bartolo, 2017).

**Figure 1:**
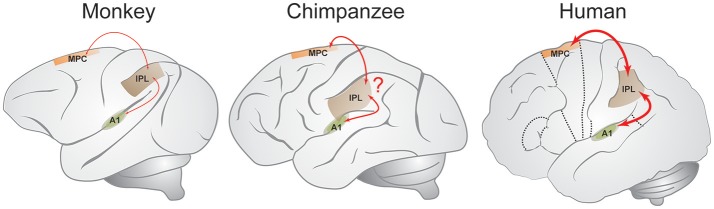
The gradual audiomotor evolution (GAE) hypothesis. The GAE hypothesis suggests connections between medial premotor cortex (MPC), inferior parietal lobe (IPL), and primary auditory area (A1) to be stronger in humans as compared to other primates (marked with red lines), suggesting beat-based timing to have gradually evolved. Line thickness indicates the hypothesized connection strength (Adapted from Mendoza and Merchant, 2014 and Merchant and Honing, 2014).

Based on these and related neurobiological observations (e.g., Zarco et al., 2009; Merchant et al., 2011) the *GAE* hypothesis was proposed (Merchant and Honing, 2014), arguing that the integration of sensorimotor information throughout the mCBGT circuit and other brain areas during the perception or execution of single intervals is similar in human and non-human primates, but different in the processing of multiple intervals. While the mCBGT circuit was shown to be also involved in beat-based mechanisms in imaging studies (e.g., Teki et al., 2011), direct projections from the medial premotor cortex (MPC) to the primary auditory cortex (A1) via the inferior parietal lobe (IPL) that is involved in sensory and cognitive functions such as attention and spatial sense (see Figure 1), may be the underpinning of beat-based timing as found in humans, and possibly apes. The GAE hypothesis suggests beat-based timing to be more developed in humans as opposed to apes and monkeys, and that it evolved through a gradual chain of anatomical and functional changes to the interval-based mechanism to generate an additional beat-based mechanism, instead of claiming a categorical jump from single-interval to multiple-interval abilities (i.e., rhythmic entrainment; Patel, 2006; Patel and Iversen, 2014). As such, the GAE hypothesis suggests that beat perception and entrainment have emerged gradually in primate order. This observation is in line with Rauschecker and Scott (2009) earlier suggestion that “the privileged access of the humans' auditory system to the sequential and temporal machinery of the mCBGT circuit emerged gradually in the course of evolution from precursors of the great ape lineage.” Some recent behavioral studies support such a gradual interpretation (Hattori et al., 2015; Large and Gray, 2015) suggesting at least some beat-based timing capabilities in apes that are absent in rhesus monkeys. Finally, the GAE hypothesis is in line with Patel and Iversen (2014), that argue for a causal link between auditory and motor planning regions needed for human beat perception. However, the GAE hypothesis differs from the latter proposal in that it (a) does not claim the neural circuit that is engaged in beat-based timing to be deeply linked to vocal learning, perception, and production, even if some explicit overlap between these neural circuits exists, and (b) that it gradually evolved in primates, instead of being solely present in humans as the only primate capable of vocal learning (Honing and Merchant, 2014).

However, in the current study we will not make claims about the underlying neural mechanisms, nor will we present a systematic comparative study (this is a topic of ongoing research). Instead, we will focus on whether rhesus monkeys are able to sense isochrony and/or the beat in a rhythmic stimulus.

## Current study

In the current study we adapted an auditory oddball paradigm that was previously used in humans (Bouwer et al., 2016) and that allows for testing and dissociating the contributions of beat perception and isochrony to the temporal predictability of the stimulus.

**Figure 2:**
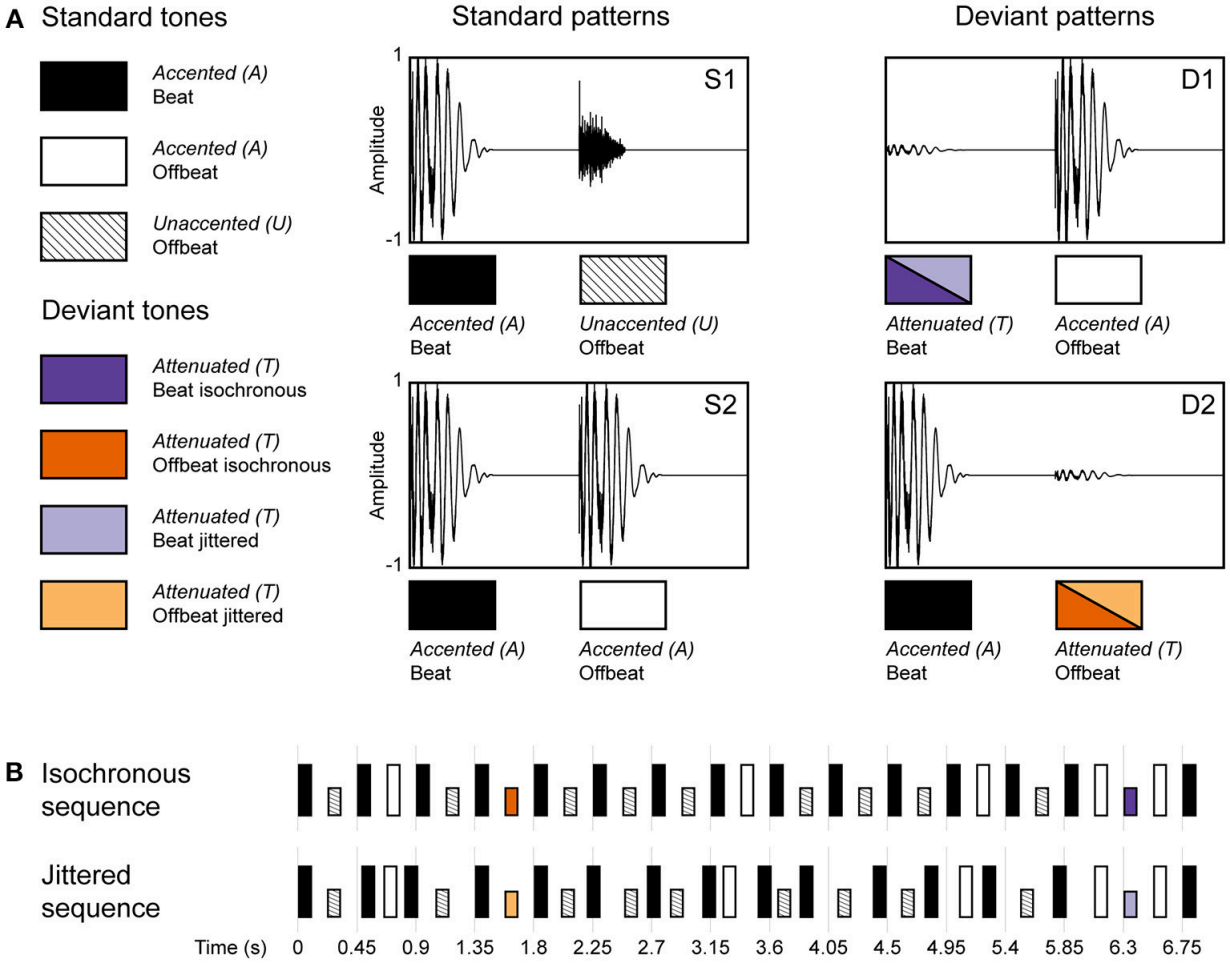
Schematic diagram of the rhythmic stimulus patterns used in the experiment. **(A)** The two standard (S1 and S2) and two deviant patterns (D1 and D2), made up of three different sounds (A, accented; U, unaccented; and T, attenuated). An accented sound could occur either on the beat or offbeat, an unaccented sound was restricted to the offbeat position. An attenuated sound was used as a deviant in two positions (beat and offbeat) and in two conditions (isochronous and jittered). **(B)** Standard and deviant sound patterns were concatenated into a single rhythmic stream (their generation is visualized as a transition network in Figure 3). Sequences in the isochronous condition had an inter-onset interval (IOI) of 225 ms, in the jittered condition these were randomly chosen from the range 150–300 ms using a uniform distribution. Deviants were always preceded and followed by an accented sound, with a fixed IOI of 225 ms in both conditions (Adapted from Bouwer et al., 2016).

We presented two rhesus monkeys (*M. mulatta*) a rhythmic sequence that was made up of a pattern of loud and soft percussive sounds such that the acoustic stimulus could *induce* a simple binary metrical structure (duple meter), with an accented beat on every other metrical position (see combinations of S1 and S2 in Figure 2). This rhythmic sequence was presented in two conditions: an *isochronous condition*, in which the sounds were presented in an isochronous fashion, using an inter-onset interval (IOI) of 225 ms, allowing a beat to be induced (i.e., one metrical level of a duple meter). And a *jittered condition*, in which the IOIs were randomly selected between 150 and 300 ms, as such disabling the perception of a beat. Furthermore, we used intensity decrements to be able to compare the ERP response to deviants in both the isochronous and jittered conditions (since omissions, as used in Honing et al., 2012, wouldn't be recognized as deviants in the jittered condition). By introducing unexpected intensity decrements (i.e., deviants) on both on the beat and offbeat positions, in both the isochronous and jittered conditions, we could probe the effect of metrical position (beat vs. offbeat), as well as the effect of isochrony (isochronous vs. jittered) on the amplitude of the MMN and the P3a. Additionally, we examined the effects of metrical position and isochrony on P1 and N1 responses to standard sounds.

This design allows for testing several hypotheses. First of all, we expected an MMN and P3a for all deviants in both the isochronous and the jittered conditions, irrespective of isochrony or metrical position. This to make sure that the auditory system of monkeys is sensitive to unexpected amplitude decrements (deviants) in a rhythmic stream. For this we predicted an effect of Type (standard vs. deviant).

Second, we did not expect to find evidence for beat perception, in line with earlier findings (Honing et al., 2012; Merchant and Honing, 2014). As such, we predicted no interaction between Position (Beat vs. Offbeat) and Isochrony (Isochronous vs. Jittered). To show beat perception, the difference between the MMN responses to deviants on the beat and offbeat in the isochronous condition should be more pronounced than the difference between the MMN responses to deviants on beat and offbeat positions in the jittered condition, in which beat perception is disabled. Without such an interaction between metrical position and isochrony, the differences between the MMN responses to different metrical positions should be interpreted as a result of sequential learning instead of beat perception (see Bouwer et al., 2016).

Third, we did expect the MMN and the P3a to be affected by the isochrony of the stimulus, with both having a higher amplitude in the isochronous condition (isochronous rhythm) as compared to the jittered condition (random rhythm). When monkeys are sensitive to the temporal regularity of the isochronous stimulus (Merchant et al., 2015b; Gámez et al., 2018) this should help in predicting the next event (i.e., increasing its temporal predictability) and enhancing its processing (Schwartze et al., 2011; Bouwer et al., 2014). Hence, it can be expected that the amplitude of the MMN and P3a in response to deviants in the isochronous condition is larger than in the jittered condition. As such, we predicted an interaction between Isochrony (Isochronous vs. Jittered) and Type (Standard vs. Deviant). In addition, we expected an inverse relationship between the amplitude of the P1 and N1 and temporal predictability. When the amplitude of the N1 and P1 is attenuated by the isochrony of the stimulus this can be used as additional evidence for isochrony perception (as was shown for humans in Schwartze et al., 2013).

## Methods

### Ethics statement

All the animal care, housing, experimental procedures were approved by the National University of Mexico Institutional Animal Care and Use Committee and conformed to the principles outlined in the Guide for Care and Use of Laboratory Animals (NIH, publication number 85–23, revised 1985). Both monkeys were monitored daily by the researchers and the animal care staff, and every second day from the veterinarian, to check the conditions of health and welfare. To ameliorate their condition of life we routinely introduced in the home cage (1.3 m^3^) environment toys (often containing items of food that they liked) to promote their exploratory behavior. The researcher that tested the animals spent half an hour interacting with the monkeys directly, giving for example new objects to manipulate. We think that this interaction with humans, in addition to the interaction that was part of the task performed, can help to reduce potential stress related to the experiment. Food and water where given ad libitum.

### Participants

Two rhesus monkeys participated in the ERP measurements. Monkey A is a 11 year old male, Monkey B a 9 year old male. Both monkeys have normal hearing. They were awake (i.e., not sedated) during the measurements, sitting in a quiet room [3 (l) × 2 (d) × 2.5 (h) m] with dimmed lighting and two loudspeakers in front of them. The ERP measurements were performed after a morning session of unrelated behavioral experiments. The animals were seated comfortably in a monkey chair where they could freely move their head, hands and feet. No head fixation was used and the EEG electrodes were attached to the monkey's scalp using tape. To ease the fixation of the electrodes, the monkey's hair on the scalp and reference ear was shaved.

### Stimuli

The stimuli were identical to those used in Bouwer et al. (2016). Rhythmic sequences were composed of two sounds that differed in timbre, intensity and duration to induce a simple binary metrical structure (duple meter) with acoustic accents. The sounds were made with QuickTime's (Apple, Inc.) drum timbres. The first sound consisted of a simultaneously sounding bass drum and hi-hat, and will be referred to as *accented* (or A for short). The second sound was a hi-hat, which was 16.6 dB softer than the accented sound and lasted 70 ms instead of 110 ms. This sound will be referred to as *unaccented* (or U for short). The deviant sound was created by attenuating the accented sound by 25 dB (using Praat software; www.praat.org), leaving timbre and duration intact. This sound will be referred to as *attenuated* (or T for short; see Figure 2).

The accented, unaccented and attenuated sounds (A, U, and T) were combined into a rhythmic stream in which 60% of the time an accented sound was followed by an unaccented sound (see S1 in Figure 2A), and 30% of the time an accented sound was followed by another accented sound (see S2 in Figure 2A), as such inducing a duple meter, with always an accented sound on the beat. In the remaining 10% of the time a deviant was inserted (the “oddball”). This was either, randomly chosen, an attenuated sound followed by an accented sound (5%; see D1 in Figure 2A) or an accented sound followed by attenuated sound (5%; see D2 in Figure 2A). An example of an isochronous and a jittered rhythmic stream are given in the Supplementary Material.

The resulting sequence was used in both the isochronous and jittered conditions (see Figure 2B). In the isochronous condition, all sounds were presented with a constant inter-onset interval of 225 ms. In this condition, the probabilistic pattern of alternating accented and unaccented sounds was expected to induce a beat (or duple meter) with an inter-beat interval of 450 ms, within the optimal range for beat perception in humans (London, 2002). Sounds in uneven positions of the sequence (including deviant D1) can be considered on the beat, while all sounds in even positions (including deviant D2) are offbeat. In the jittered condition, the inter-onset intervals were randomly distributed between 150 and 300 ms with an average of 225 ms (uniform distribution), using the same sequence as in the isochronous condition, making it impossible to induce a regular beat (London, 2002; Honing, 2012). However, the inter-onset interval just before and after a deviant tone was kept constant at 225 ms. This was done to make the acoustic and temporal context in which a deviant occurs, identical between both conditions.

In addition, four additional constraints were applied to the construction of the sound sequences. To optimize the possibility of inducing a beat in the isochronous condition, S2 (containing two consecutive accented sounds) was never presented more than once in a row, and only a maximum of four consecutive S1 patterns was allowed. Furthermore, a deviant on the beat (D1) was always preceded by an accented sound offbeat (S2), ensuring the acoustical context to be identical for all deviants. Finally, at least five standard patterns occurred between two deviant patterns. For schematic examples of both the isochronous and jittered sequences, see Figure 2B. Note that the D1 and D2 are referred to as on the beat or offbeat in both conditions for comparison, while this can only be perceived as such in the isochronous condition.

**Figure 3:**
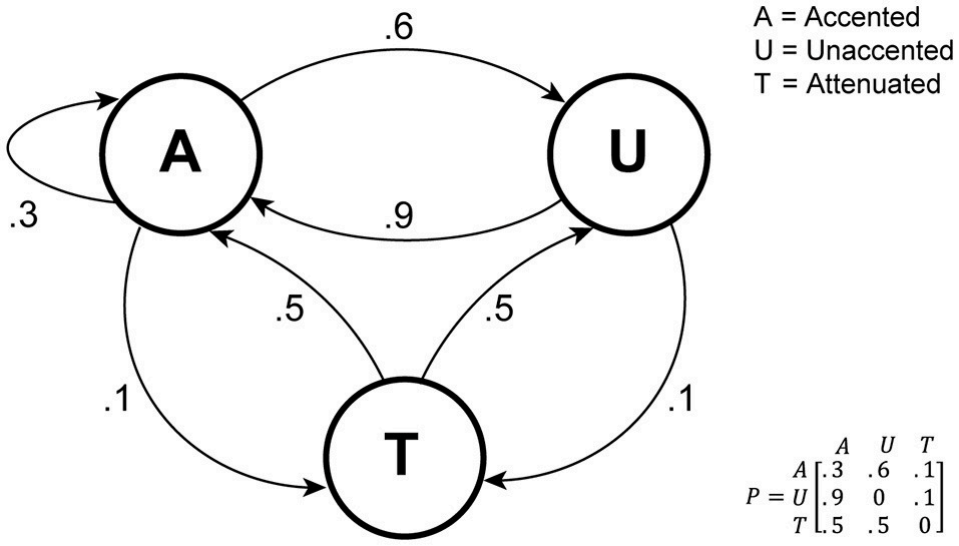
Rhythmic sequence generation visualized as a transition network. As noted in the text, this representation is a simplification in that it does not include certain constraints on which patterns can follow each other.

The statistical properties of the sequences used are visualized in Figure 3 as a transition network, with the three basic sounds A, U, and T as nodes. Note that this is a simplification in the sense that it does not include the four constraints mentioned above.

### Procedure

Stimuli were presented in blocks consisting of 3 isochronous and 3 jittered sequences (1,300 pattern, i.e., 2,600 sound events, per sequence), in randomized order. One block (of 6 sequences) lasted 9 min and 45 s, separated by a silent interval of about 15 s. Isochronous and jittered blocks were presented in semi-random order, with a maximum of two blocks from the same condition following each other. This resulted in a total session length of 60 min, one session per day.

Sound stimuli were presented through 2 loudspeakers placed 1.1 meters away from the subject (and 1 meter apart from each other). The sound intensity measured at the subject position was 80 dB SPL. The monkeys participated in one recording session per day, to a total of 14 sessions for Monkey A and 12 sessions for Monkey B. All measurements were completed in about 5 weeks per monkey.

Overall, the design of the study was identical to the unattended condition presented in Bouwer et al. (2016), except that in the human study participants watched a silent video with subtitles, whereas in the current study monkeys were not given any visual stimuli to focus on.

### EEG recording and analysis

**Figure 4:**
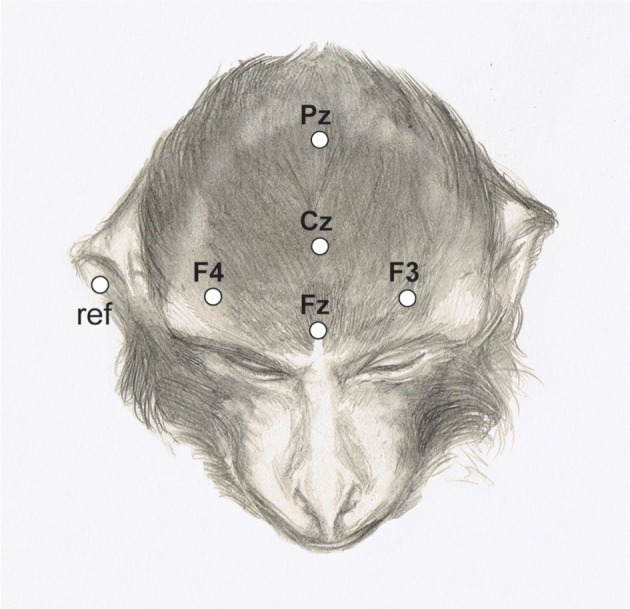
Electrode positions marked on the head of a rhesus monkey. (Adapted from Honing et al., 2012).

The EEG was recorded from electrodes (Grass EEG electrodes; #FS-E5GH-60) attached to five scalp positions (Fz, Cz, Pz, F3, F4) according to the 10–20 system (see Figure 4).

The electrodes were connected to a Tucker-Davis Technologies (TDT) headstage (#RA16LI) for low impedance electrodes. This headstage was connected to a TDT RA16PA preamplifier, which in turn was connected to a TDT RZ2 processor. RZ2 was programmed to acquire the EEG signals with a sampling rate of 498.25 Hz and the bandpass filters were set at 0.01–100 Hz.

All electrodes were attached using Ten20 Conductive EEG Paste and medical tape, and were referenced to the right ear (fleshy part of the pinna). In the offline analysis, a 0.1–30 Hz band-pass FIR filter (Kaiser-window) was applied.

### MMN and P3a

For the analyses of MMN and P3a, epochs of −150 to 300 ms were extracted for the four deviant patterns (D1_i_, D1_j_, D2_i_, D2_j_; with subscripts referring to isochronous and jittered conditions). Epochs of the same length were extracted for accented sounds from the standards in the isochronous condition, both on the beat (from S1, but only if preceded by S2) and offbeat (from S2). Thus, the acoustic context preceding all sounds that were used in the analysis of the MMN and P3a was identical (i.e., they were preceded by an accented sound at −225 ms). Epochs were baseline corrected using the average voltage of the 150 ms prior to the onset of the tone and averaged to obtain ERPs for each condition and monkey. We obtained difference waves by subtracting the ERP responses to the accented sounds from the standard patterns from the ERP responses to the deviant tones at the same position (beat or offbeat). Epochs that exceeded ±300 μV amplitude were excluded from the statistical analysis. The number of epochs accepted for analysis are given in Tables 2, 3.

**Table 2:** MMN.

**Monkey A**				
	**Beat**		**Offbeat**	
	**D (*****n*** = **2,695)**	**S (*****n*** = **13,567)**	**D (*****n*** = **2,708)**	**S (*****n*** = **11,683)**
Isochronous	−0.37 (0.27)	1.60 (0.12)	−0.001 (0.27)	1.34 (0.13)
	(n = 2,719)	(n = 13,567)	(n = 2,724)	(n = 11,683)
Jittered	0.30 (0.27)	1.60 (0.12)	0.50 (0.28)	1.34 (0.13)
**Monkey B**				
	**Beat**		**Offbeat**	
	**D (*****n*** = **2,125)**	**S (*****n*** = **10,544)**	**D (*****n*** = **2,104)**	**S (*****n*** = **9,176)**
Isochronous	−0.58(0.31)	1.10 (0.14)	−0.38 (0.31)	0.95 (0.15)
	(n = 2,250)	(n = 10,544)	(n = 2,273)	(n = 9,176)
Jittered	0.39 (0.31)	1.10 (0.14)	0.51 (0.29)	0.95 (0.15)

*Mean amplitudes of standard and deviant waves at Cz in the isochronous and jittered conditions for Monkey A and Monkey B. Mean amplitudes (μV) are indicated with SE values in parentheses. S, values for standard stimuli; D, values for deviant stimuli; n, number of epochs. The time window used in the statistical analyses is 57–87 ms for Monkey A and 95–125 ms for Monkey B*.

**Table 3:** P3a.

**Monkey A**				
	**Beat**		**Offbeat**	
	**D (*****n*** = **2,695)**	**S (*****n*** = **13,567)**	**D (*****n*** = **2,708)**	**S (*****n*** = **11,683)**
Isochronous	0.78 (0.21)	−1.51 (0.10)	1.23 (0.23)	−1.72 (0.11)
	(n = 2,719)	(n = 13,567)	(n = 2,724)	(n = 11,683)
Jittered	−0.06 (0.22)	−1.51 (0.10)	0.03 (0.22)	−1.72 (0.11)
**Monkey B**				
	**Beat**		**Offbeat**	
	**D (*****n*** = **2,125)**	**S (*****n*** = **10,544)**	**D (*****n*** = **2,104)**	**S (*****n*** = **9,176)**
Isochronous	0.21 (0.27)	−0.95 (0.13)	0.32 (0.28)	−0.96 (0.13)
	(n = 2,250)	(n = 10,544)	(n = 2,273)	(n = 9,176)
Jittered	0.58 (0.27)	−0.95 (0.13)	0.35 (0.24)	−0.96 (0.13)

*Mean amplitudes of P3a at Cz in the isochronous and jittered conditions. Mean amplitudes (μV) are indicated with SE values in parentheses. Number of epochs: n between brackets. The time window used in the statistical analyses is 126–176 ms for Monkey A and 178–228 ms for Monkey B*.

We defined the amplitude of the MMN as the average amplitude from a 30 ms window (relatively small to avoid overlap with the N1) centered around the average peak latency across conditions on Cz (electrode that was shown to be maximally indicative of MMN in rhesus monkeys; Honing et al., 2012; Gil-da-Costa et al., 2013). The MMN peaked at Cz on average at 72 ms for Monkey A and at 110 ms for Monkey B. See the caption of Table 2 for the time windows used in the statistical analyses.

We defined the amplitude of the P3a as the average amplitude from a 50 ms window centered around the average peak latency across conditions on Cz (electrode that was shown to be maximally indicative of P3a in rhesus monkeys; Gil-da-Costa et al., 2013). The P3a peaked at Cz on average at 151 ms for Monkey A and at 203 ms for Monkey B. See the caption of Table 3 for the time windows used in the statistical analyses.

### P1 and N1

For the analysis of P1 and N1 epochs of −100 to 300 ms were extracted for accented standards on the beat and offbeat, including only those standards that were preceded by an accented sound. A filter of 5–75 Hz was applied to eliminate slow drift (as is commonly used in studying these components in humans; see Schwartze et al., 2013; Bouwer and Honing, 2015). Hence, baseline correction was not needed. Epochs that exceeded ±300 μV amplitude were excluded from the statistical analysis.

The peak amplitude of both the P1 and N1 was defined on the average of the waveforms collapsed over conditions, using 20 ms window centered around the average peaks at Cz (electrode that was also shown in other studies to be maximally indicative of P1 and N1 in monkeys; see Itoh et al., 2015). A 20 ms window was chosen around the average P1 and N1 peaks to avoid overlap between the P1 and N1 windows. Table 4 shows the average amplitudes for all four conditions, the number of epochs accepted for analysis, and the time windows used for statistical analyses.

**Table 4:** P1 and N1.

**Monkey A**				
	**Beat**		**Offbeat**	
	**P1 (*****n*** = **13,598)**	**N1 (*****n*** = **13,598)**	**P1 (*****n*** = **11,707)**	**N1 (*****n*** = **11,707)**
Isochronous	0.83 (0.12)	−2.24 (0.12)	0.64 (0.13)	−2.37 (0.13)
	(n = 13,494)	(n = 13,494)	(n = 11,774)	(n = 11,773)
Jittered	1.28 (0.13)	−3.08 (0.13)	1.65 (0.14)	−2.96 (0.14)
**Monkey B**				
	**Beat**		**Offbeat**	
	**P1 (*****n*** = **10,734)**	**N1 (*****n*** = **10,734)**	**P1 (*****n*** = **9,307)**	**N1 (*****n*** = **9,307)**
Isochronous	2.85 (0.10)	−1.72 (0.10)	2.96 (0.11)	−1.62 (0.11)
	(n = 11,224)	(n = 11,224)	(n = 9,750)	(n = 9,750)
Jittered	2.27 (0.10)	−1.74 (0.10)	2.43 (0.11)	−1.59 (0.11)

*Mean amplitudes of P1 and N1 at Cz in the isochronous and jittered conditions. Mean amplitudes (μV) are indicated with SE values in parentheses. Number of epochs: n between brackets. The time window used in the statistical analyses for Monkey A is 20–40 ms for the P1 and 40–60 ms for the N1. For Monkey A the time window used is 18–38 ms for the P1 and 40–60 ms for the N1*.

### Statistical analysis

The amplitudes extracted from the difference waves were entered into ANOVAs with factors Position (Beat vs. Offbeat), Isochrony (Isochronous vs. Jittered), and Type (Standard vs. Deviant) for the MMN and P3a analyses, and the factors Position (Beat vs. Offbeat), Isochrony (Isochronous vs. Jittered) for the P1 and N1 analyses. Partial eta squared ($\eta_{p}^{2}$) was used as a measure of effect size. All statistical analyses were conducted in SPSS (Version 22).

## Results

### MMN and P3a

**Figure 5:**
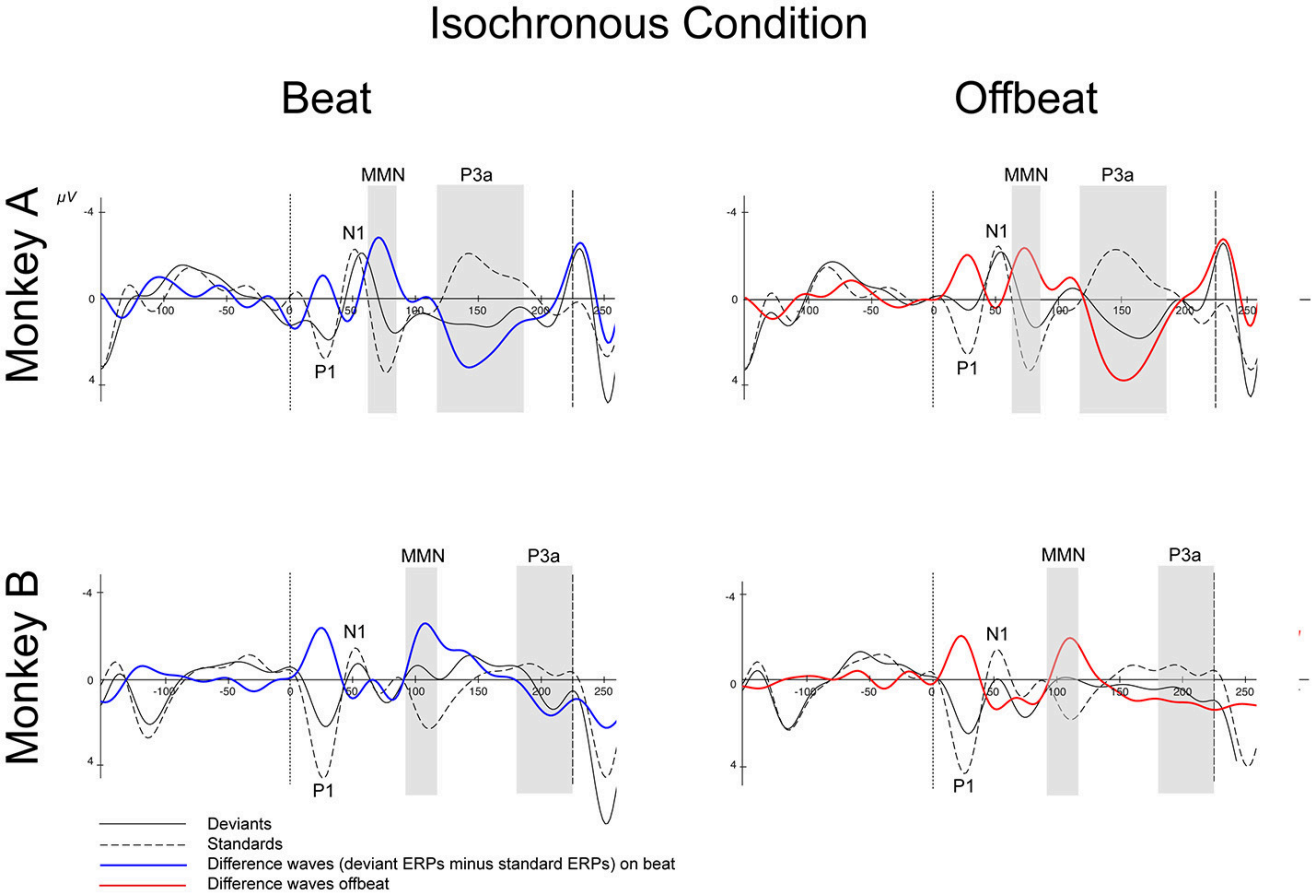
Event-related potentials at Cz for the isochronous condition. Deviants are marked with solid black lines, standards with dotted black lines. Difference waves (deviant ERPs minus standard ERPs) on the beat are represented with solid blue lines, offbeat with solid red lines, for Monkey A and Monkey B. The gray-shaded areas indicate the time windows used in the statistical analyses of the MMN and P3a. Dotted vertical lines indicate the start of the sound event, dashed vertical lines indicate the onset of the next sound event (at 225 ms). See Table 2 for details on time ranges used.

**Figure 6:**
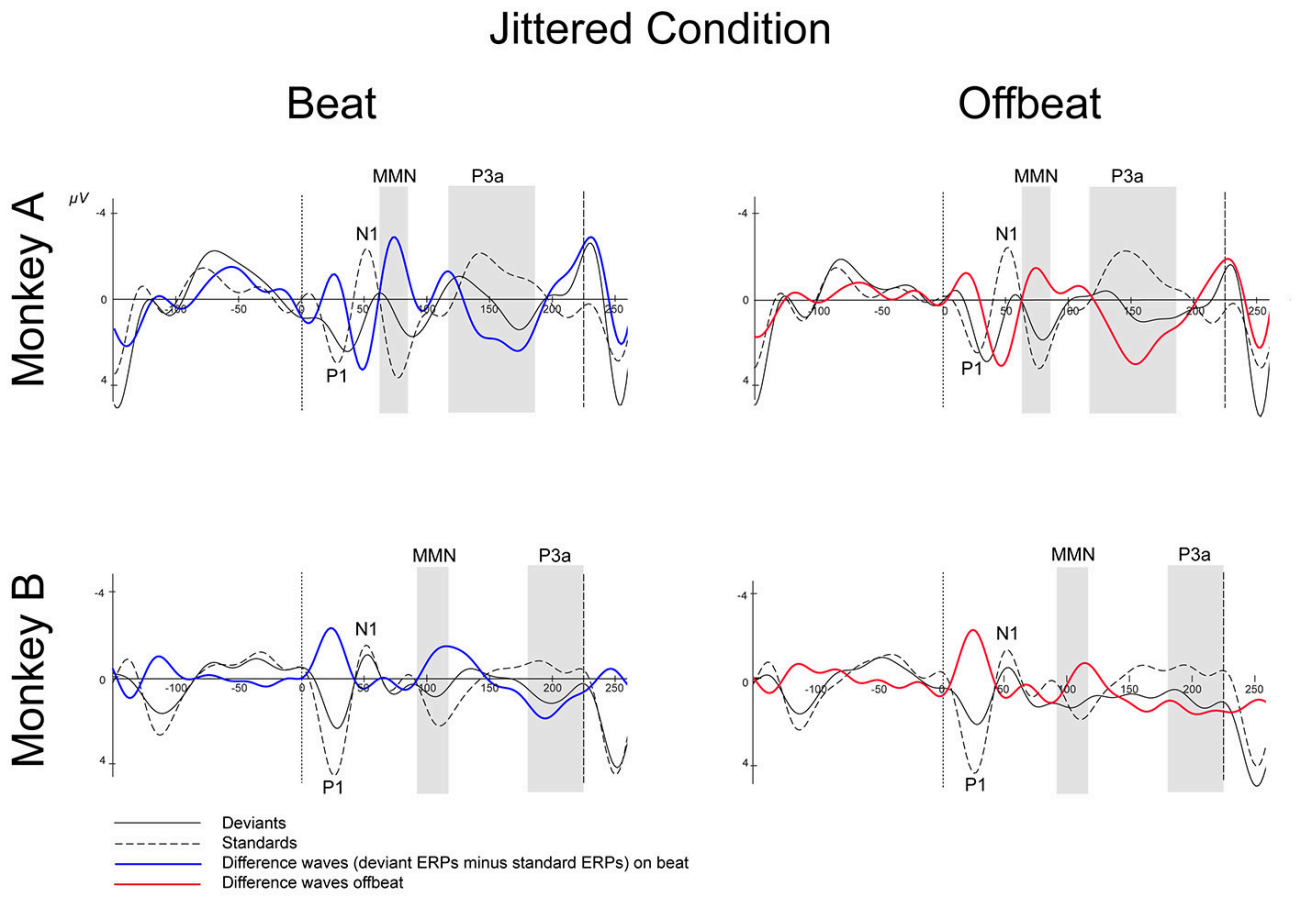
Event-related potentials at Cz for the jittered condition. Deviants are marked with solid black lines, standards with dotted black lines. Difference waves (deviant ERPs minus standard ERPs) on the beat are represented with solid blue lines, offbeat with solid red lines, for Monkey A and Monkey B. The gray-shaded areas indicate the time windows used in the statistical analyses of the MMN and P3a. Dotted vertical lines indicate the start of the sound event, dashed vertical lines indicate the onset of the next sound event (at 225 ms). See Table 3 for details on time ranges used.

Figures 5, 6 show that, for both monkeys, the ERPs elicited by the standard (dotted lines) and the deviant (solid lines) are different, with peaks in the interval 60–110 ms for the MMN and 100–250 ms for the P3a, consistent with earlier studies (see Table 1).

For both monkeys the ANOVA with factors Type (Standard vs. Deviant), Isochrony (Isochronous vs. Jittered) and Position (Beat vs. Offbeat) revealed a significant main effect of Type {Monkey A: [*F*_(1, 61345)_ = 83.790, *p* < 0.0005, $\eta_{p}^{2}$ = 0.001]; Monkey B: [*F*_(1, 48191)_ = 38.906, *p* < 0.0005, $\eta_{p}^{2}$ < 0.001]}, showing that the evoked brain response to deviants was significantly negative as compared to the standard in all conditions. In addition, there was an interaction of Isochrony and Type {Monkey A: [*F*_(1, 61345)_ = 3.896, *p* < 0.048, $\eta_{p}^{2}$ = 0.0005]; Monkey B: [*F*_(1, 48191)_ = 7.819, *p* < 0.005, $\eta_{p}^{2}$ < 0.0005]} showing an effect of isochrony on the size of the MMN, being larger in the isochronous condition. This suggests a sensitivity to the isochrony of the stimulus. However, no effects of Position or an interaction between Position and Isochrony were found. Hence, there is no support for beat perception. See Table 2 for all MMN measurements and **Figure 8** for a summary.

With regard to the P3a there is a significant main effect of Type {Monkey A: [*F*_(1, 61345)_ = 292.069, *p* < 0.0005, $\eta_{p}^{2}$ = 0.005]; Monkey B: [*F*_(1, 48191)_ = 76.793, *p* < 0.0005, $\eta_{p}^{2}$ < 0.002]} and an interaction of Isochrony and Type {Monkey A: [*F*_(1, 61345)_ = 17.032, *p* < 0.0005, $\eta_{p}^{2}$ = 0.0005]; Monkey B: n.s.}. For Monkey A there was also a significant interaction between Position and Type {Monkey A: [*F*_(1, 61345)_ = 3.884, *p* < 0.049, $\eta_{p}^{2}$ < 0.0005]}. Note that this is in a direction opposite to what was found in humans (Bouwer et al., 2016), which makes this result difficult to interpret. See Table 3 for all P3a measurements and **Figure 8** for a summary.

### P1 and N1

**Figure 7:**
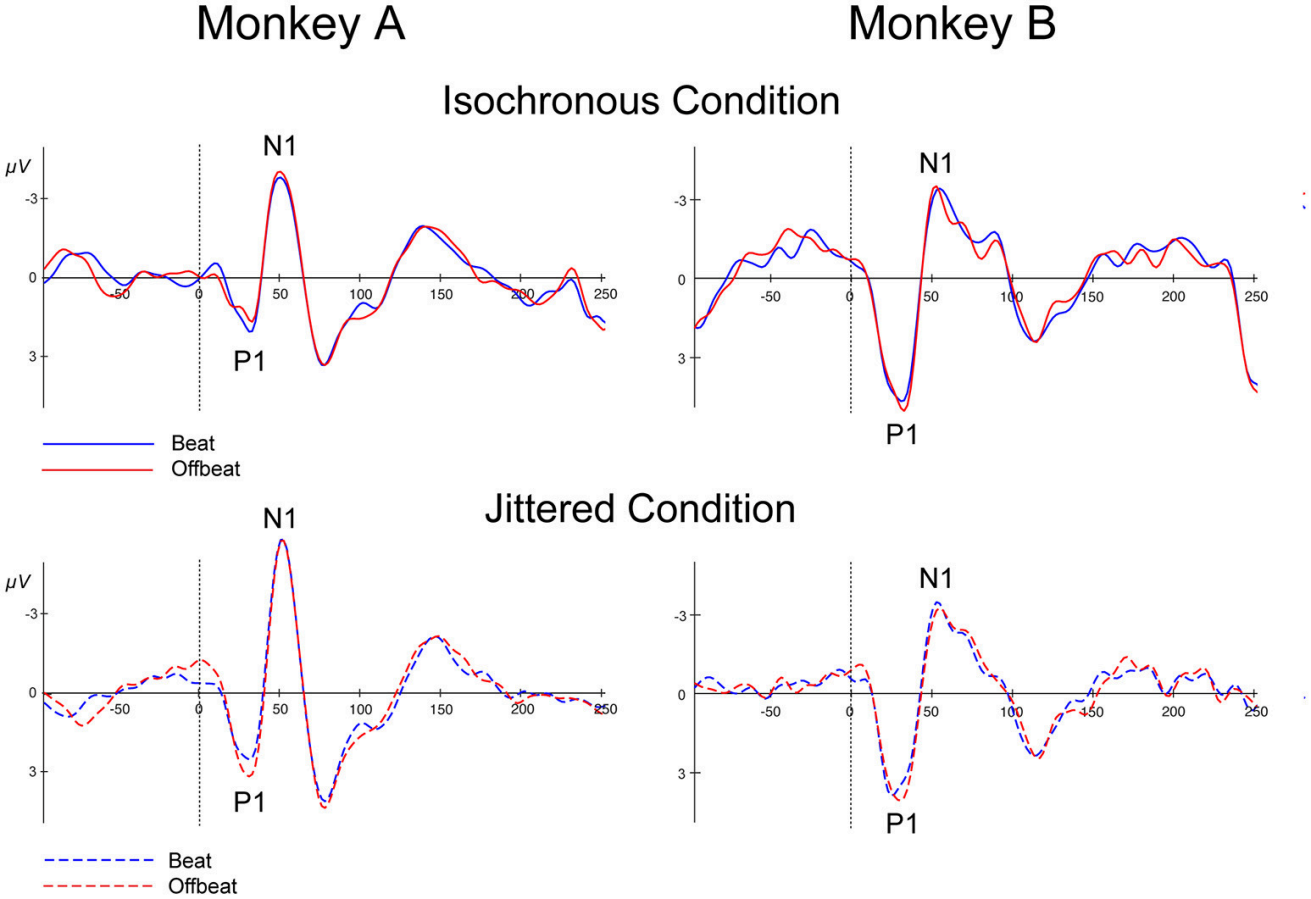
Event-related potentials at Cz for all conditions. Standards in the isochronous condition **(Top)** and jittered condition **(Bottom)** for Monkey A and Monkey B. Standards on the beat are marked in blue and standards offbeat are marked in red.

Figure 7 shows the P1 and N1 responses to the standard in the isochronous condition (solid lines) and jittered condition (dotted lines) for both monkeys. Table 4 shows the average amplitudes for all four conditions and the time windows used for statistical analyses.

For both monkeys the ANOVA with factors Isochrony (Isochronous vs. Jittered) and Position (Beat vs. Offbeat) revealed a significant main effect of Isochrony for both the P1 {Monkey A: [*F*_(1, 50572)_ = 31.306, *p* < 0.0005, $\eta_{p}^{2}$ = 0.001]; Monkey B: [*F*_(1, 41014)_ = 27.912, *p* < 0.0005, $\eta_{p}^{2}$ = 0.001]} and the N1 {Monkey A: [*F*_(1, 50572)_ = 30.822, *p* < 0.0005, $\eta_{p}^{2}$ = 0.001]; Monkey B: n.s.} showing that the P1 is significantly different in the jittered as compared to the isochronous condition for both monkeys, and the N1 only for Monkey A. However, in the case of the P1 the direction of the effect is different for both monkeys. In Monkey A both the P1 and the N1 are larger for the jittered as compared to the isochronous sounds. This is in line with the idea that unpredictable stimuli receive more processing, hence resulting in larger amplitude responses (Schwartze et al., 2011, 2013). However, in Monkey B the effect of Isochrony on the P1 response is in the opposite direction, with larger responses to the sounds in the isochronous as opposed to in the jittered sequences. As such, this weakens the interpretation that the P1 is indicative of predictability. Additionally, we found an interaction between Isochrony and Position in Monkey A {[*F*_(1, 50572)_ = 4.585, *p* < 0.032, $\eta_{p}^{2}$ < 0.0005]}. However, the latter is in the opposite direction as compared to humans (Baldeweg, 2007; Costa-Faidella et al., 2011) and hence hard to interpret. All other effects were not significant.

## Discussion

In the current study we examined the role of beat perception and isochrony perception in two rhesus monkeys using the same stimuli in an oddball paradigm that was previously used in humans (Bouwer et al., 2016). Similar to humans, we found MMNs to all deviants as opposed to standards in both the isochronous and jittered conditions. From this we conclude that the monkey brain is sensitive to unexpected amplitude decrements (deviants). This is in line with earlier studies that used either omissions (Honing et al., 2012) or intensity deviants (Gil-da-Costa et al., 2013).

**Figure 8:**
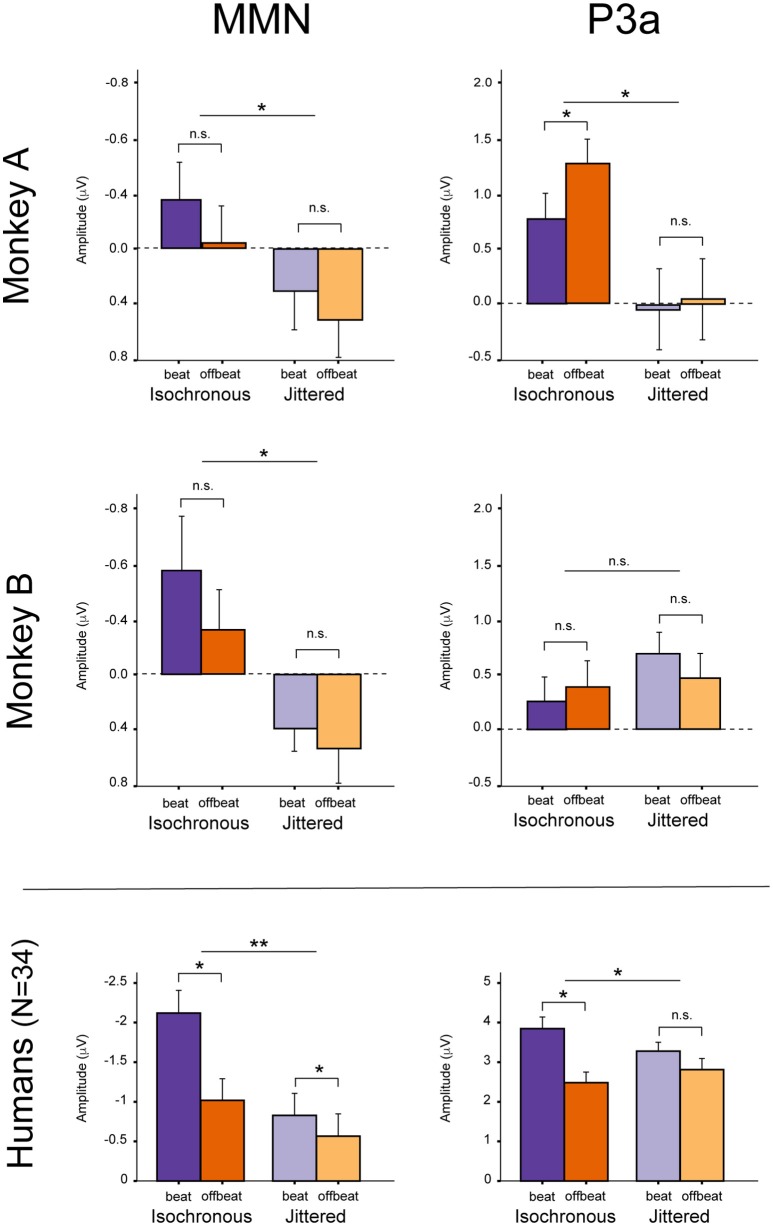
Summary of the results. MMN and P3a in Monkey A **(Top)** and Monkey B **(Middle)**. For comparison, the **(Bottom)** shows the mean results for humans (*N* = 34), unattended condition (see Table 1 in Bouwer et al., 2016). Note that the y-axes are different for the monkey and human data. Error bars represent one standard error of the mean. *Significant interaction.

However, and contrary to what was found in humans (Bouwer et al., 2016), there are no significant differences in the MMN between deviants in beat positions as opposed to deviants in an offbeat position, neither in the isochronous condition nor the jittered condition (i.e., no effect of metrical position, nor an interaction with regularity; see Figure 8). So while in humans the beat appears to modulate the amplitude of the MMN, in monkeys there was not such an effect. This suggests the absence of beat perception in monkeys, in line with an earlier study (Honing et al., 2012).

With regard to the P3a, Monkey A did show an interaction between Type and Isochrony, but this was small and in the opposite direction as compared to humans (see Figure 8). This makes the latter result difficult to interpret. The effect might be a result of overlapping components (e.g., an interference with the strong response to the next onset; marked with dashed lines in Figures 5, 6), as such affecting the overall amplitude of the ERP signal. It could also be a result of attentional fluctuations that are modulating the P3a, as was shown in human studies (cf. Bouwer et al., 2016). Future work should manipulate attention to make sure whether and how attention influences rhythm perception.

By contrast, both monkeys appear to be sensitive to the isochrony of the stimulus, as is supported by an overall larger MMN response to deviants in the isochronous as opposed to the jittered condition (see Figure 8). Note however, that we cannot make the claim that monkeys detect isochrony (hence the use of the term “sense”). It could well be that the monkey brain is able to sense isochrony, but that the monkey is not aware of this, as was recently shown in two beat-deaf humans (Mathias et al., 2016). The latter study suggested the P3a to be associated with conscious perception or “awareness,” comparable to what is observed in tone deaf humans (Peretz, 2016).

With regard to the N1 and P1 the results are mixed and not consistent between Monkey A and Monkey B. While the attenuation of the N1 of Monkey A is in support for isochrony perception, the P1 is in opposite directions for the two monkeys. This difference could again be caused by attention, which was not controlled for in this study. As such we have to be cautious in our interpretation of the P1 and N1 in the standards, and will base our conclusions mainly on the MMN in response to the deviants (see summary in Table 5 and main results in Figure 8).

**Table 5:** Summary of the results.

	**Deviants**		**Standards**	
	**MMN**	**P3a**	**P1**	**N1**
Monkey A	^*^	^*^	^*^	^*^
Monkey B	^*^	n.s.	^–^	n.s.

The effect of isochrony on MMN and P3a (enhancement), and temporal predictability on P1 and N1 (attenuation).

* significant; n.s., non-significant;

– *significant in opposite direction*.

In short, the ERPs of both monkeys appear to be influenced by isochrony, but not by the induced beat. Where humans show a clear interaction between metric position and isochrony (Bouwer et al., 2016), and as such show evidence for beat perception, the current results provide no evidence for beat perception in rhesus monkeys. Hence, the hypothesis that beat-based timing is common to all animals (Darwin, 1871; Wilson and Cook, 2016) is not supported by these results.

While the underlying neural mechanism (be it interval-based timing or otherwise; Teki et al., 2011, 2016) is as yet unclear, we take this result as further support for the GAE hypothesis (Merchant and Honing, 2014). The GAE hypothesis suggests beat-based timing to be a result of bidirectional bottom-up and top-down interactions between the auditory and motor areas of the brain, including the mCBGT circuit and parietal areas such as the IPL (see Figure 1), connections that are quite developed and efficient in humans and that emerged gradually in the course of evolution from precursors of the great ape lineage (Rauschecker and Scott, 2009; Honing and Merchant, 2014; Merchant and Honing, 2014; Morillon et al., 2014; Merchant and Yarrow, 2016).

Parts of the audiomotor circuit, including areas such as the putamen in the basal ganglia or the parietal cortex, also process information from the dorsal stream of visual processing (Kimura, 1992; Merchant et al., 2004). Therefore, it could well be that the areas involved in the strong visuomotor coupling in monkeys partially overlap with the beat-entrainment audiomotor system, in line with the predictive and entrainment abilities of monkeys with visual stimuli (Takeya et al., 2017; Gámez et al., 2018). Thus, where humans show a preference for auditory metronomes, monkeys have a clear preference for visual stimuli (Gámez et al., 2018). Applying the current paradigm to the visual modality (e.g., with different intensity flashes rather than different intensity tones) might be able to show beat-entrainment for visual stimuli.

Gámez et al. (2018) also provides evidence that monkeys can predictively entrain to an isochronous metronome, even when it accelerates or decelerates. These findings suggest that the beat-based mechanisms of macaques might not be as restricted as previously thought (Patel, 2014). Thus, it is crucial that future experiments focus on finding the limits in beat perception and entrainment capabilities of monkeys, using gradually more complex levels of metrical periodicity in their stimuli (Schwartze and Kotz, 2015; Bouwer et al., 2016).

Finally, and as predicted by the GAE hypothesis, we expect beat-based timing in the auditory modality to be present in some rudimentary form in apes (not monkeys). Two recent behavioral studies support this interpretation (Hattori et al., 2015; Large and Gray, 2015) for a chimpanzee (*P. troglodytes*) and a bonobo (*Pan paniscus*). Applying the same oddball paradigm to a chimpanzee (cf. Ueno et al., 2008) might find further support for the GAE hypothesis (Merchant and Honing, 2014). In addition, the paradigm could be extended to other species, for example, pigeons, rodents, cats and dogs (e.g., Nelken and Ulanovsky, 2007; Howell et al., 2012; Schall et al., 2015; Harms et al., 2016), where the MMN can be measured to probe the potentially shared capabilities of isochrony perception and/or beat perception.

While musicality is likely made up of many components, it appears to be good strategy to start with a focus on core aspects, like beat perception (cf. Honing, 2018b). The core aspects of musicality are well suited for comparative studies, both cross-cultural and cross-species, and the nature and extent of their presence in non-human animals have attracted considerable debate in the recent literature. These recent discussions, combined with the availability of suitable experimental techniques for tracking these phenomena in human and non-human animals, make this a timely and feasible enterprise. Of course, we need to remain cautious about making claims on music-specific modes of processing until more general accounts have been ruled out. It still has to be demonstrated that the constituent components of musicality, when identified, are indeed domain specific. In contrast, the argument that music is a human invention (Patel, 2018) depends on the demonstration that the components of musicality are not domain specific, but each cognitively linked to some non-musical mental ability. So while there might be quite some evidence that components of musicality overlap with non-musical cognitive features (Patel, 2018), this is in itself no evidence against musicality as an evolved biological trait or set of traits. As in language, musicality could have evolved from existing elements that are brought together in unique ways, and that system may still have emerged as a biological product through evolutionary processes, such as natural or sexual selection. As such there is no need for musicality to be modular or show a modular structure. Alternatively, based on the converging evidence for music-specific responses along specific neural pathways, it could be that brain networks that support musicality are partly recycled for language, thus predicting more overlap than segregation of cognitive functions. In fact, this is one possible route to test the Darwin-inspired conjecture that musicality precedes music and language (Honing, 2018a).

## Author contributions

HH and FB conceived and designed the experiments. HM and LP performed the experiments. FB and HH analyzed the data. HH, FB, and HM wrote the paper.

### Conflict of interest statement

The authors declare that the research was conducted in the absence of any commercial or financial relationships that could be construed as a potential conflict of interest.

## Footnotes

^1^*Musicality*, in all its complexity, can be defined as a natural, spontaneously developing set of traits [designed for the perception and production of music] based on and constrained by our cognitive abilities and their underlying biology. As such, *music*, in all its variety, can be defined as a social, and cultural construct based on that very musicality (Honing, 2018a).

## Supplementary material

Audio 1 Example of a rhythmic pattern used in the isochronous condition. [1_isochronous.wav].

Audio 2 Example of a rhythmic pattern used in the jittered condition. [2_jittered.wav].
